# Mixed Cu–Mn Oxide Catalysts for Solvolysis of Technical Lignin

**DOI:** 10.1021/acssuschemeng.4c09666

**Published:** 2025-02-18

**Authors:** Davey F. de Waard, Panos D. Kouris, Michael D. Boot, Emiel J. M. Hensen

**Affiliations:** Laboratory of Inorganic Materials and Catalysis, Department of Chemical Engineering and Chemistry, Eindhoven University of Technology, P.O. Box 513, Eindhoven 5600 MB, Netherlands

**Keywords:** lignin, catalytic solvolysis, ethanol, hydrotalcite, CuMn

## Abstract

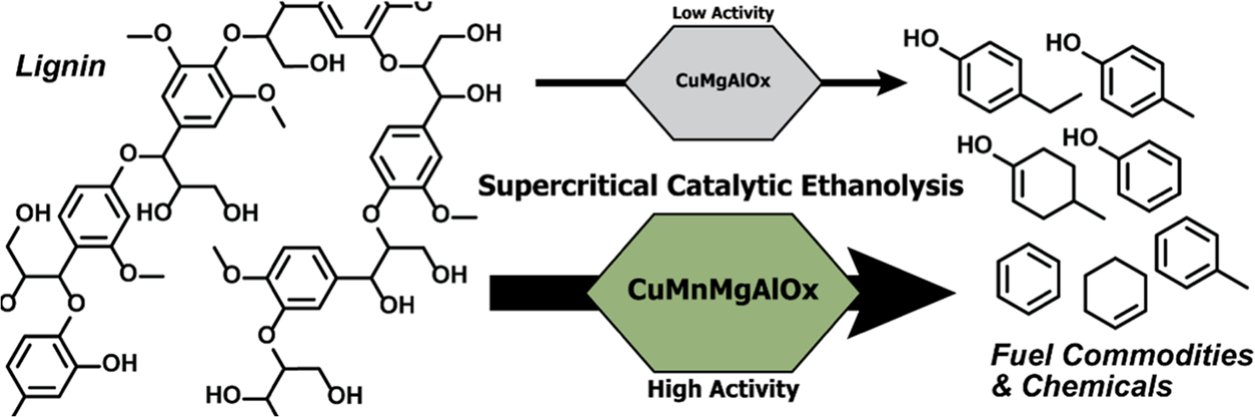

With the rising demand for fuel and the societal shift toward sustainable resources, lignin emerges as a prime feedstock. Lignin is mainly composed of aromatic compounds linked within a complex matrix and holds significant potential as a source of renewable aromatics. Technical lignin, the most abundant form of lignin, is often degraded due to harsh biomass pretreatment processes. Cu_20_MgAlO_*x*_ porous mixed oxide (CuPMO) is an efficient catalyst to help solvolyze technical lignin. Here, we demonstrate the promotion of such mixed oxides with Mn toward improving both the yield of monomers and solubilized lignin oil. The promotion was highest at a Cu/Mn ratio of unity, resulting in a 2-fold increase in monomer extraction compared to the benchmark CuPMO. The Mn-doped catalyst produced more saturated products. Simultaneously, solvent consumption decreased with increasing Mn content. X-ray diffraction (XRD) and X-ray photoelectron (XPS) analyses revealed the formation of a Cu–Mn spinel oxide. The proximity of Cu and Mn in this precursor facilitated the reduction of Mn through hydrogen spillover from Cu^0^ formed during catalyst reduction during heating in the reaction mixture. The observed increase in saturated products, coupled with enhanced lignin solvolysis, highlights the superior hydrogenation capability of the CuMnMgAlO_*x*_ catalyst for the solvolysis of technical lignin.

## Abstract 2

A Mn-doped Cu_20_MgAlO_*x*_ catalyst in supercritical ethanol was found to boost lignin monomer yield and hydrogenation over a Cu-only catalyst.

## Introduction

Lignin can play an important role in the future production of fuel commodities and chemicals as an abundant source of renewable aromatics.^1,2^ Lignin is a biopolymer consisting of three different types of aromatic alcohol units linked in various ways into a cross-linked rigid material.^3,4^ Depending on how the lignin is extracted from biomass, different qualities of lignin can be obtained. For example, Kraft pulping aimed at extracting high-quality cellulose from lignocellulose requires harsh conditions, which strongly degrades lignin. This also holds for soda pulping, which chemically resembles Kraft pulping with the difference that sodium sulfide is not used. The strong degradation of lignin stems from the cleavage of labile β–O–4 bonds, resulting in smaller fragments with reactive groups that condense into stronger C–C bonds. Further upgrading the resulting technical lignin into monomers and oligomers is more difficult. To overcome this problem, lignin can be extracted from the biomass in approaches termed lignin-first refining.^5^ As lignin is most often regarded as a byproduct in industry, most of the lignin available is technical lignin.^6^

Various strategies exist to depolymerize lignin, often using catalysts containing noble metals.^1^ There is also attention to replacing such expensive metals with earth-abundant metals. A porous CuMgAlO_*x*_ mixed metal oxide, derived from a hydrotalcite precursor, has drawn significant attention as a cheap and effective catalyst for lignin upgrading. Ford and co-workers showed high yields for the depolymerization of organosolv lignin to combined C2–C6 alcohols and hydrogenated aromatics in supercritical methanol.^7^ The CuPMO catalyst also effectively reformed methanol into H_2_ and CO_2_. H_2_ was used to hydrodeoxygenate the oxygen-containing moieties in lignin and further hydrogenate olefinic and aromatic groups. Yan et al. demonstrated the use of a combined MoC/CuMgAlO_*x*_ catalyst for the production of aromatic monomers from kraft lignin. Huang et al. used the same CuMgAlO_*x*_ catalyst in the ethanolysis of technical soda lignin.^8^ Catalyst development resulted in an optimum composition of ca. 20% Cu and a M^2+^/M^3+^ ratio of 4.^9^ Huang et al. also detailed the roles of the various components in the CuMgAlO_*x*_ catalysts. Hydrogenolysis reactions were found to be catalyzed by reduced Cu metal, with H_2_ for the hydrogenolysis and hydrogenation reactions being in situ produced by the conversion of ethanol into acetaldehyde on basic MgO_*x*_ sites. Reactive lignin fragments are further converted through acid and basic sites on the catalyst surface. Combining Cu with MgO basic sites catalyzes the formation of higher alcohols and esters through Guerbet and Tishchenko reactions, which involve the condensation of aldehydes into heavier products. A higher activity in Guerbet and esterification reactions results in less repolymerization of the lignin fragments,^9^ which was ascribed to the scavenging of formaldehyde, a reaction intermediate involved in the oligomerization of phenolic fragments, in these reactions.^10^ The combination of Cu and Lewis acidic Al_2_O_3_ sites was found to alkylate the phenolic monomers using the ethanol solvents and alcohol products formed during the reaction. Alkylation stabilizes phenolic fragments against recondensation, improving the overall extraction efficiency of monomers. Thus, CuMgAlO_*x*_ mixed oxides are versatile catalysts with various types of active sites for the depolymerization of lignin.

Here, we explore the further modification of these CuPMO catalysts by introducing first-row transition metals, aiming at improved performance in the solvolysis of technical lignin. A screening study identified Mn as a suitable promoter for CuMgAlO_*x*_ in the solvolysis of soda lignin. The state of Mn in CuMgAlO_*x*_ and its role in catalytic solvolysis was characterized in detail. The introduction of Mn in the hydrotalcite and the mixed metal oxide derived thereof led to a higher CuO dispersion with an optimum activity reached at a Cu/Mn ratio of 1. Overall, a three times higher monomer yield was achieved in the catalytic solvolysis of soda lignin at the complete dissolution of lignin for the optimum CuMnMgAlO_*x*_ catalyst compared to the CuMgAlO_*x*_ benchmark.

## Methods

### Catalyst Preparation

A series of Mn-containing CuMgAlOx mixed oxide catalysts were obtained by calcination of hydrotalcite precursor materials, in which base transition metals were doped. Hydrotalcites were prepared by a coprecipitation approach involving a constant M^2+^/M^3+^ ratio of 4, which was previously found to be optimal for lignin conversion.^9^ Another set of materials was prepared after identifying Mn as the most promising promoter. At a constant Cu loading of 20 wt %, the Cu/Mn ratio was varied by choosing the amounts of Cu^2+^, Mg^2+^, Mn^2+^, and Al^3+^ such that a constant M^2+^/M^3+^ ratio of 4 was retained. A typical preparation involved the addition of 3.72 g Cu(NO_3_)_2_·2.5H_2_O, 12.31 g Mg(NO_3_)_2_·6H_2_O, 7.50 g Al(NO_3_)_3_·9H_2_O, and 4.01 g Mn(NO_3_)_2_·4H_2_O to 100 mL Milli-Q water. This solution was added dropwise to a 150 mL solution of Na_2_CO_3_ (5.09 g). To maintain the pH of the solution at 9.5–10, a 2 M NaOH solution was added dropwise (typically 50–80 mL during 45 min). The slurry was kept at 60 °C under vigorous stirring during this preparation. After the addition of the metal precursor solution, the slurry was aged for 24 h at 60 °C, while vigorously stirring. The coprecipitation step was performed under a protective atmosphere of flowing Ar to protect Mn from oxidizing. The resulting suspension was filtered, and the solid residue was washed until the filtrate reached a pH of 7. The solid was dried overnight at 110 °C and sieved to a particle size below 200 μm. The mixed metal oxide was obtained by calcinating the hydrotalcite in air for 6 h at 460 °C (heating rate 2 °C/min). A MgAlO_*x*_ reference sample was prepared in the same way without Cu. Samples are denoted by Cu_*x*_Mn_*y*_, where *x* and *y* refer to the wt % of Cu and Mn, respectively.

### Catalyst Characterization

Inductively coupled plasma atomic emission spectrometry (ICP-AES) was used for elemental analysis using a Spectro Ciros CCD ICP optical emission spectrometer. The samples were dissolved in an equimolar mixture of H_2_O and H_2_SO_4_. Powder X-ray diffraction (XRD) was performed on a Bruker D2 Phaser with a Cu K-alpha radiation source. XRD patterns were recorded in the 5–80° range with 0.02° increments using a step time of 0.2 s. N_2_ physisorption was performed on a Micromeritics Tristar 3000 system. Prior to physisorption measurements, samples were degassed at 300 °C for 3 h. X-ray photoelectron spectroscopy (XPS) was carried out on a K-alpha XPS spectrometer (Thermo Scientific) equipped with a monochromatic Al Kα (1486.6 eV) X-ray source. The spot size was 400 μm with pass energies of 200 and 50 eV for survey and element scans, respectively. The C 1s feature of adventitious carbon with a binding energy of 284.8 eV was used for charge correction. N_2_O chemisorption was performed in a stainless-steel reactor tube coupled to a mass spectrometer for online gas analysis. Before each experiment, the catalyst was reduced in a H_2_ flow at 350 °C for 1 h. Afterward, N_2_O chemisorption experiments were performed by switching to a 10 mL/min flow of 1 vol % N_2_O in He. Chemisorption experiments were performed at 30 °C to avoid bulk oxidation.^11^

### Catalytic Activity Measurements

Catalytic solvolysis of lignin was performed in a 100 mL stainless steel high-pressure autoclave from Parr Instrument Company. Typically, the autoclave was filled with 1 g of feedstock (soda lignin, P1000) and 0.5 g of catalyst with 40 mL ethanol. As an internal standard 20 μL of *n*-dodecane was added to the reaction mixture. The reactor was sealed and purged 5 times with N_2_ to ensure the removal of residual air. After leak testing, the pressure was increased to either 10 or 30 bar with, respectively, N_2_ or H_2_. The reactor was heated to 340 °C within 1 h while stirring at 500 rpm. After reaching the reaction temperature, the reactor was left for 4 h. The reaction was stopped by removing the reactor from the heating block. Upon reaching a temperature of 200 °C, the reactor was quenched in an ice–water mixture. A liquid sample was taken from the reaction mixture for gas chromatography-flame ionization detector/mass spectrometry (GC-FID/GC-MS) analysis. The rest of the reaction mixture was subjected to a workup procedure designed to separate light and heavy lignin fragments through tetrahydrofuran (THF) fractionation, as described earlier by Huang et al.^9^

### Product Analysis

The liquid-phase products were analyzed on a Shimadzu 2010 GC-MS system equipped with a RTX-1701 column (60 m x 0.25 mm × 0.25 μm), a flame ionization detector (FID), and a mass spectrometer detector. The products were identified using NIST11 and NIST11s MS libraries, while their concentrations were determined by the effective carbon number using n-dodecane as the internal standard. The products were grouped into four groups, namely (i) oxygen-containing aromatics, (ii) oxygen-free aromatics, (iii) oxygen-containing saturated compounds, and (iv) oxygen-free saturated compounds. The yields of the THF soluble/insoluble lignin, monomers, and char were calculated using eqs 1–4. Ethanol conversion products were quantified in the same way as the lignin fragments
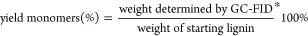
1
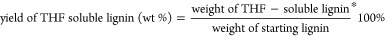
2
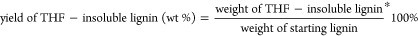
3
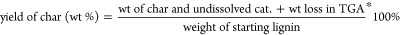
4

Gaseous products were analyzed using an Interscience Compact GC system equipped with Molsieve 5A and Porabond Q columns, each equipped with a thermal conductivity detector (TCD), and an Al_2_O_3_/KCl column with an FID. Quantification of the gaseous products was done against a calibration mixture containing known quantities of C1–C4 hydrocarbons, CO/CO_2_, and H_2_.

NMR spectra of the starting lignin, lignin residue, and bio-oils were recorded on a Bruker 400 MHz spectrometer. For the analysis of the starting lignin and the lignin residues, 100 mg of solids was dissolved in 0.6 mL of DMSO-*d*_6_. ^1^H–^13^C HSQC NMR spectra were obtained with a phase-sensitive gradient-edited HSQC program (gHSQCAD). The main parameters were 0–12 ppm in F2 (^1^H) with 1024 data points, 0–200 ppm in F1 (^13^C) with 256 data points, and a 2 s relaxation delay. The residual DMSO solvent peak was used as an internal reference (δ_C_ = 39.5 ppm; δ_H_ = 2.50 ppm). Data was processed using the MestReNova software. For the assignments of the spectra features, we followed the literature.^8,10,12^

Size exclusion chromatography (SEC-GPC) was done using a Shimadzu HPLC system with PLGel mixed-C and mixed-D columns (Agilent) placed in series and a ultraviolet–visible (UV–vis) detector (238 nm). The column was calibrated using an universal polystyrene standard. Analysis was carried out at 25 °C using THF as the eluent (1 mL/min). Samples were prepared at a concentration of 2 mg/mL THF. Prior to injection all samples were filtered with a 0.45 μm membrane filter.

## Results and Discussion

### Effect of Dopant on CuMgAlO_*x*_

Hydrotalcite is a layered double hydroxide (LDH) of the general formula Mg_6_Al_2_CO_3_(OH)_16_·4H_2_O. The structure of LDHs is based on that of brucite Mg(OH)_2_, where layers of hydroxide anions coordinating with Mg and Al cations are separated by either water or carbonate groups. Calcination of hydrotalcite leads to mixed metal oxides with tunable textural and chemical properties.^13^ These properties and the surface reactivity of the porous metal oxides obtained by calcination can be further modified by replacing Mg^2+^ and Al^3+^ in the LDH precursor with other metal cations. Earlier work has demonstrated that a Cu-porous metal oxide (CuPMO) derived from Cu, Mg, and Al is a suitable catalyst for lignin depolymerization. We prepared a set of LDH precursors with an M^2+^/M^3+^ ratio of 4 and a Cu content of 20 wt % by coprecipitation.^9,14^ The hydrotalcite precursors varied in the type of metal dopant, where besides Cu, Mg, and Al, a fourth metal was introduced. Mg^2+^ and Al^3+^ were partly replaced by M^2+^ (Mn, Co, Ni, and Zn) and M^3+^ (Cr, Fe, and Ga) dopants, respectively. The modified CuPMO catalysts were obtained by calcination at 460 °C and used as catalysts for the depolymerization of soda lignin in supercritical ethanol at 300 °C.

**Figure 1:**
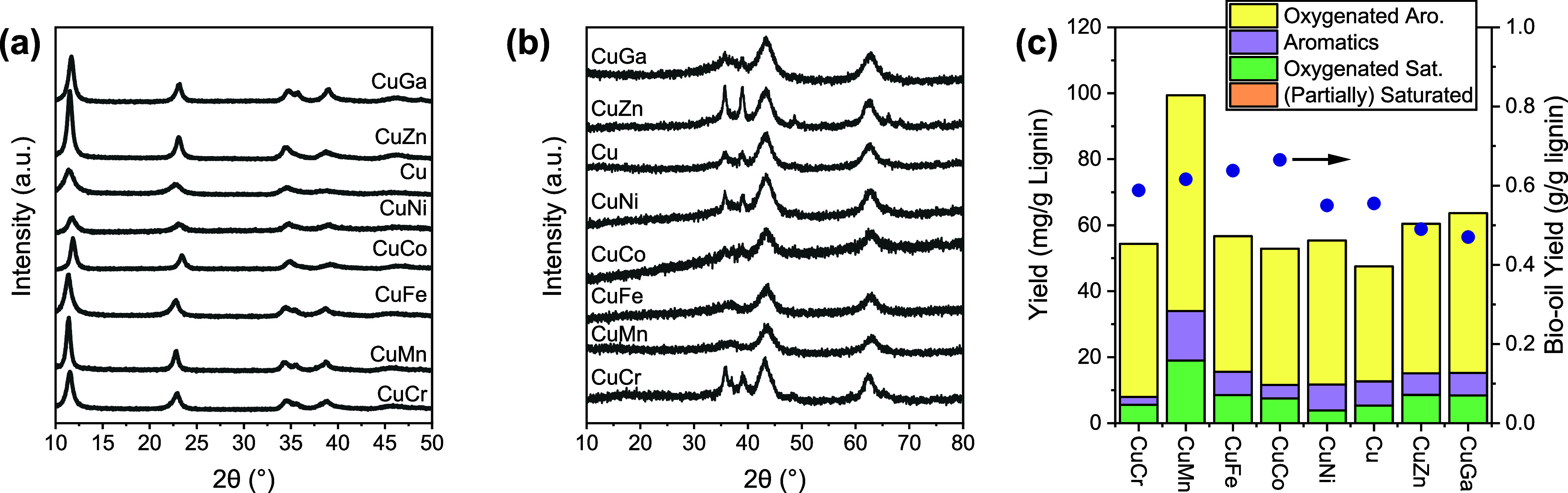
XRD patterns of (a) hydrotalcite precursors and (b) calcined products, (c) monomer and bio-oil yield of catalytic solvolysis P1000 lignin (Conditions: 1 g P1000, 0.5 g Cat., Ethanol, 300 °C, 10 bar N_2_, 4 h hold).

Figure 1 shows the XRD patterns of the hydrotalcite precursors and the calcined products. After coprecipitation, only the hydrotalcite structure was observed irrespective of the dopant. Some variation in the peak positions can be related to the difference in ionic radii, resulting in different lattice spacings. The hydrotalcite structure was lost upon calcination. The main crystalline phases in the calcined product can be assigned to CuO and MgO. For the materials doped with Co, Fe, and Mn, only crystalline MgO was observed. When the precursor was doped with Zn, Ni, and Cr, the CuO peaks were stronger than those in the dopant-free CuPMO sample. The varying CuO peak intensity points to differences in CuO dispersion. The materials obtained with Co, Fe, and Mn dopants have the highest CuO dispersion. These three samples also showed the highest bio-oil yield in the catalytic solvolysis of P1000 lignin, which was significantly higher than the bio-oil yield of CuPMO. The other dopants showed a similar (CuNi) or lower (CuZn and CuGa) bio-oil yield, likely because of the lower CuO dispersion. While the variation in monomer yield was small among the doped samples and with respect to the CuPMO sample, CuMn showed a much higher monomer yield. Based on these screening results, we selected Mn-modified CuPMO for further optimization.

**Figure 2:**
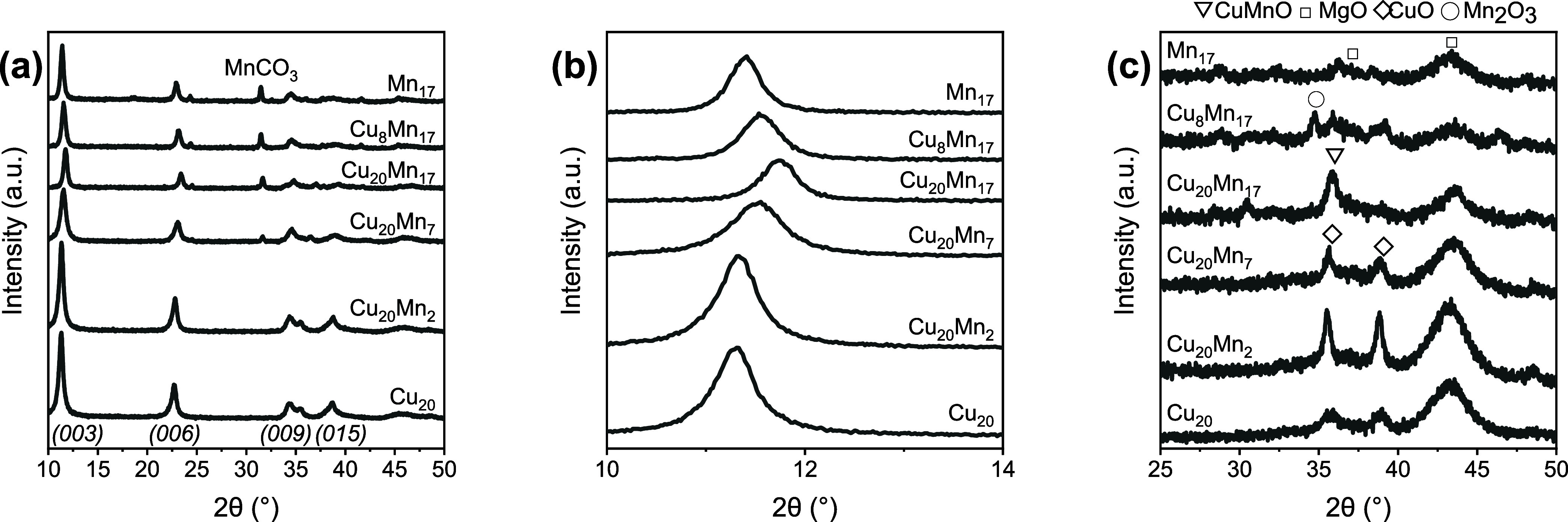
XRD pattern of Mn-doped hydrotalcite (a) magnification of the (003) reflection (b) and the calcined catalyst (c).

The Mn loading was optimized by preparing a new set of CuMnPMO samples. Figure 2 shows the XRD patterns of these samples, including those of Cu_20_PMO and Mn_17_PMO references. One subset of these samples had a Cu loading of 20 wt % and an increasing Mn loading ranging from 0 to 17 wt %. Another subset had a Mn loading of 17 wt % and varying Cu loading, ranging from 0 to 20 wt %. The XRD patterns in Figure 2a show hydrotalcite as the main phase in the freshly prepared samples. From the shift of the (003) reflection (zoom in Figure 2b), it can be concluded that Mn is present in the hydrotalcite structure, replacing the larger Mg^2+^ ion. With increasing Mn content, the hydrotalcite lattice was observed to contract. Similarly, starting from Mn_17_MgAlO_*x*_, replacing Mg^2+^ with Cu^2+^ reduced the unit cell volume. The XRD patterns indicate the presence of MnCO_3_ at high Mn loading. It cannot be excluded that the sample also contained small MnCO_3_ particles at lower Mn loading, escaping XRD detection. Figure 2c shows the XRD patterns of the calcined catalysts. As expected, the calcined samples no longer exhibit the hydrotalcite structure, with only CuO and MgO as crystalline phases. The intensity of the CuO reflections decreased with increasing Mn loading. In Cu_20_Mn_17_PMO, the reflections at 35 and 39° assigned to CuO were replaced by a single diffraction line at 36°, which can be assigned to a mixed spinel oxide of Cu and Mn.^15,16^

Table 1 collects the physicochemical properties of the CuMnPMO samples. The metal loadings were slightly lower than expected, which can be explained by the absorption of atmospheric CO_2_ and H_2_O after hydrotalcite precipitation.^9^ The collapse of the hydrotalcite by calcination typically increases the surface area together with the development of significant mesoporosity.^17,18^ The Brunauer–Emmett–Teller (BET) surface areas of the Mn-doped samples were well above 150 m^2^/g. The lower surface area of the Cu_20_Mn_17_PMO sample is likely due to the lower crystallinity of the parent hydrotalcite.

**Table 1:** Physicochemical Properties of CuMnMgAlO_*x*_ Mixed Metal Oxides

catalyst	BET (M^2^/g)	*V* (cm^3^/g)	*d* (nm)	Cu (wt %)	Mg (wt %)	Al (wt %)	Mn (wt %)
MgAlO*_x_*	229	0.62	15.2		45.8	12.7	
Cu_20_MgAlO*_x_*	182	0.56	15.3	20.8	31.9	11.0	
Cu_20_Mn_2_MgAlO*_x_*	203	0.75	19.1	20.6	30.7	10.9	1.78
Cu_20_Mn_7_MgAlO*_x_*	183	0.80	20.7	19.9	27.3	10.5	6.9
Cu_20_Mn_17_MgAlO*_x_*	131	0.45	17.0	18.5	21.2	9.8	16.0
Cu_8_Mn_17_MgAlO*_x_*	192	0.55	13.2	8.0	27.8	10.7	17.5
Mn_17_MgAlO*_x_*	195	0.53	13.9		36.1	10.0	16.3

**Figure 3:**
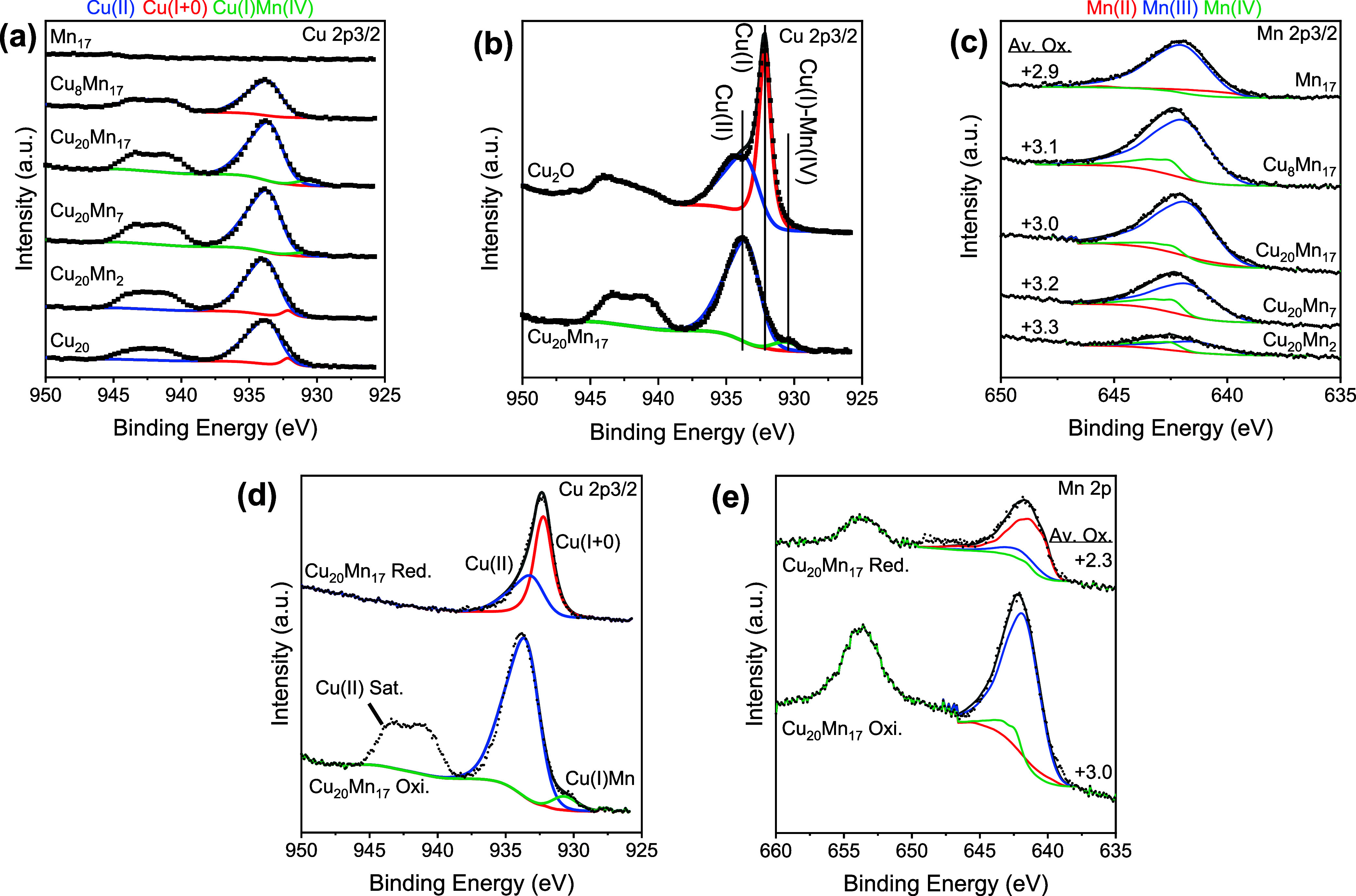
XPS spectra of Mn-doped CuPMO: Cu 2p (a, b, d), Mn 2p (c, e) regions for as-synthesized and reduced catalysts.

Figure 3 shows relevant XPS spectra of the calcined samples. The Cu 2p and Mn 2p spectra were fitted using fitting models described in the literature.^19,20^ The spectra were corrected for charging by setting the C 1s binding energy of adventitious carbon to 284.8 eV. In the Cu 2p spectra, two 2p_3/2_ contributions were observed, a major one at ∼933.7 eV and a minor one at ∼932.0 eV, assigned to respectively Cu^2+^ and Cu^+^/Cu^0^. Given their calcined state, the samples most likely contain a combination of Cu^2+^ and Cu^+^. Cu LMM spectra were of insufficient resolution to resolve the presence of Cu^0^. With increasing Mn loading, the Cu^+^ contribution disappeared, and a new Cu 2p_3/2_ contribution became visible with a binding energy of ∼930.5 eV (zoom in Figure 3b). This contribution has a lower 2p_3/2_ binding energy compared to Cu^+^ in Cu_2_O. The most likely explanation is the presence of tetrahedral Cu^+^ in a CuMn spinel oxide.^21−24^ The presence of such spinel oxide was also indicated by XRD. The specific interactions between Cu and Mn in this spinel oxide would result in lower binding energies of the Cu^2+^ and Cu^+^ core levels.^16^ However, only the Cu^+^ binding energy was shifted in our case. It is found that Cu^+^ integrates into the spinel structure first, while Cu^2+^ species are predominantly present in separate CuO phase.

Figure 3c shows the Mn 2p spectra of the Mn-doped samples. Three contributions can be observed in the Mn 2p_3/2_ region, at ∼641.3, ∼ 641.8, and ∼642.6 eV, representing Mn^2+^, Mn^3+^, and Mn^4+^ oxidation states, respectively. Based on the peak areas, an average oxidation state of Mn of +2.9 was calculated for the Mn_17_PMO reference. Mn was more oxidized in the Mn-doped CuPMO samples. The highest oxidation state was found for the Cu_20_Mn_2_PMO sample (+3.3). The Mn oxidation state slightly decreased with increasing Mn loading, with the lowest value found for Cu_20_Mn_17_PMO (+3). A lower Cu loading led to a higher average Mn oxidation state. These findings suggest that Cu and Mn are close to each other in the calcined samples. The XRD pattern of the sample with the highest Mn loading shows the formation of Cu–Mn spinel oxide.

Figure 3d and e show the XPS spectra of the Cu_20_Mn_17_PMO sample suspended in ethanol after reduction in a batch reactor at a temperature of 340 °C and a (cold) pressure of 30 bar H_2_. Afterward, the catalyst was filtered and transferred to a vacuum oven for drying. The transfer was done in the wet state to prevent as much as possible exposure to air. The Cu 2p_3/2_ spectra of the used sample indicate the predominant presence of Cu^+/0^ with only a small amount of Cu^2+^ remaining. XRD confirms the presence of metallic Cu^0^. Nevertheless, it cannot be excluded that Cu^+^ is also present in the activated samples. The Mn 2p spectra contain a main contribution of Mn^2+^ and a small amount of Mn^3+^ (Figure 3e). The average Mn oxidation state is +2.3, which implies that part of Mn is also reduced to a lower oxidation state than in the precursor. The Mg 1s spectra also showed shifts between calcined and reduced Cu_20_PMO and Cu_20_Mn_17_PMO samples shifted to higher binding energies upon reduction (Figure S1), resulting in a similar effect as shown in Figure 3d,e.

**Figure 4:**
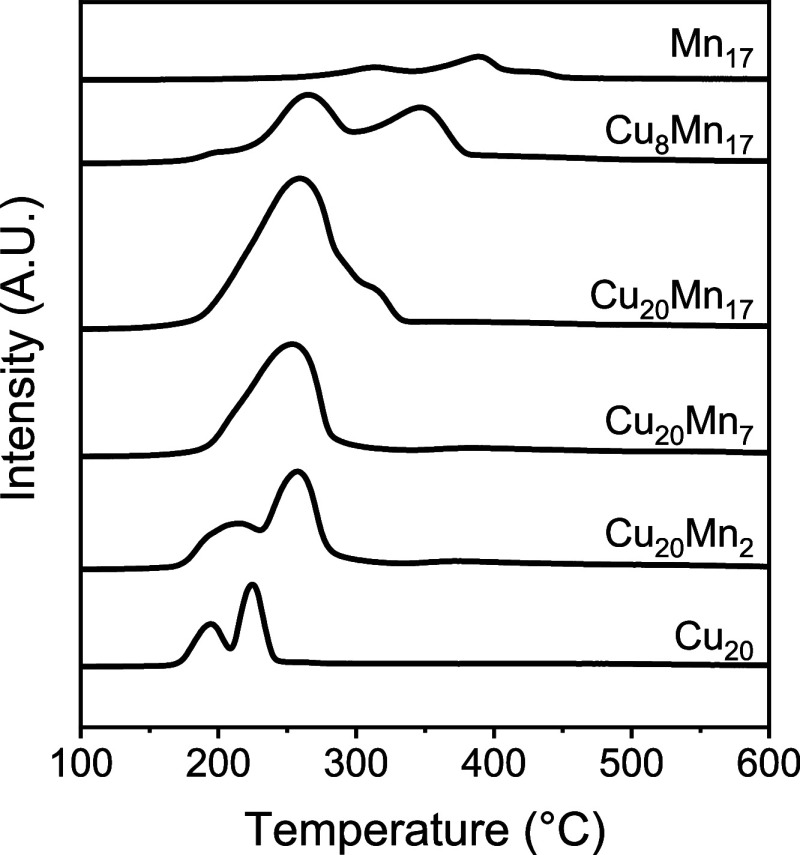
H_2_-TPR traces of calcined samples.

Figure 4 shows H_2_-TPR traces of the calcined sample, while Table 2 collects the resulting H_2_-uptake values. The Cu_20_PMO reference sample exhibited two distinct reduction features at 195 and 225 °C. These peaks can be attributed to the reduction of Cu^2+^ to Cu^+^ and Cu^+^ to Cu^0^, respectively. The Mn_17_PMO reference sample displayed three peaks at 312 °C, 388 °C, and 431 °C, which can be assigned to the stepwise reduction of Mn^4+^ to Mn^3+^, Mn^3+^ to Mn^2+/3+^, and Mn^2+/3+^ to Mn^2+^, respectively. The reduction temperatures of Mn in the Mn-doped CuPMO samples were lower than those for the Mn_17_PMO sample. For Cu_8_Mn_17_PMO, two distinct peaks were observed at 264 and 347 °C, which can be assigned to the reduction of Cu and Mn. For Cu_20_Mn_17_PMO, a single reduction peak was obtained at 259 °C with a weak shoulder at 310 °C. The results show that the reduction of Mn is facilitated by the presence of Cu, where metallic Cu, formed at lower temperatures, facilitates Mn reduction by hydrogen spillover. The onset reduction of Cu in the PMO consequently increased to higher temperatures.

**Table 2:** H_2_ and N_2_O Consumption Determined from H_2_-TPR and N_2_O Decomposition Experiments, and the Relative Cu Dispersion Derived from N_2_O Decomposition

catalyst	H_2_ uptake (mmol/g)	N_2_O consumption (μmol/g)	Cu dispersion (%)^a^
Cu_20_MgAlO_*x*_	2.4	125	7.6
Cu_20_Mn_2_MgAlO_*x*_	5.3	123	7.6
Cu_20_Mn_7_MgAlO_*x*_	6.8	156	9.9
Cu_20_Mn_17_MgAlO_*x*_	9.3	178	12.2
Cu_8_Mn_17_MgAlO_*x*_	5.8	85.5	13.6
Mn_17_MgAlO_*x*_	1.7	^+^	

a Cu dispersion is defined as the number of surface atoms divided by total Cu atoms, determined by N_2_O titration.

N_2_O chemisorption was performed to probe the metallic Cu surface. Table 2 presents the N_2_O consumption and the corresponding Cu dispersion, while Figure S6 displays a representative MS profile obtained during N_2_O chemisorption. Typically, first only N_2_ was observed in the reactor effluent due to the oxidation of metallic Cu by N_2_O. Once the Cu metal surface is saturated as Cu_2_O, N_2_O is observed. In this way, we determined the Cu dispersion of the reduced Cu_20_MgAlO*_x_* catalyst to be 7.6%. The Cu dispersion increased with increasing Mn content. The highest Cu dispersion for the catalysts containing ∼20 wt % Cu was 12.2% for Cu_20_Mn_17_MgAlO*_x_*. The increasing Cu dispersion is likely due to the interactions between Cu and MnO.^25^ At a lower Cu content, Cu_8_Mn_17_MgAlO_x_ had a slightly higher Cu dispersion of 13.6%.

### Catalytic Solvolysis of Technical Lignin

The samples were evaluated for their catalytic performance in the solvolysis of P1000 soda lignin in supercritical ethanol at 340 °C and a N_2_ pressure of 10 bar. The main results regarding the yield of monomers, oligomers, and solvent conversion are given in Table 3. A comparison with literature is shown in Table S1. The β–O–4 content of the starting soda lignin, determined with HSQC NMR, was 7%. An estimation of the monomer yield based on the cleavage of only β–O–4 bonds predicts a low monomer yield of ca. 3%.^26,27^ Earlier work performed by Huang et al.^8−10^ extensively studied the solvolysis of soda lignin using a CuMgAlO_*x*_ (CuPMO) catalyst. In the present work, a Mn-doped CuPMO was investigated for the depolymerization of soda lignin (P1000) in supercritical ethanol. The most effective PMO, comprising 20 wt % Cu and an M^2+^/M^3+^ ratio of 4, achieved a monomer yield of 36%. The fraction of soda lignin solubilized, referred to as THF-soluble lignin residue, was 69%. Typically, a small fraction of the lignin remains strongly adsorbed to the catalyst surface, denoted as THF-insoluble lignin residue, which accounts for 6% of the initial lignin in the reference case. The current study adopted a similar approach to depolymerizing lignin to assess the benefit of Mn doping. The benchmark Cu_20_PMO catalyst yielded 14.8% monomers, with THF-soluble and THF-insoluble amounts of 91.7 and 18.4%, respectively. The differences in yield with the previous work by Huang et al. are most likely due to the use of a different batch of soda lignin. The addition of Mn led to an increase in the monomer yield, and the increase was larger at higher Mn loading. At a relatively low Mn loading in Cu_20_Mn_2_PMO, the monomer yield was 24.1 with 110% lignin solubilization. Lignin solubilization values higher than 100% can be explained by the alkylation of some of the products or esterification/etherification reactions with ethanol, as demonstrated in the work of Huang et al.^10^ The highest monomer yield of 33.9% was obtained with the Cu_20_Mn_17_PMO sample with 85.5% lignin solubilization. Notably, char formation was not observed during the catalytic treatment of the lignin with all PMO catalysts.

**Table 3:** Product Distribution Obtained from Lignin Solvolysis Using Cu/Mn PMO Catalysts (1 g P1000 Lignin, 0.5 g Catalyst, 40 mL Ethanol, 340°C, 10 Bar N_2_, 4 h)

		lignin products					ethanol products		
entry	catalyst	monomers [%]	THF-soluble LR^a^ [%]	THF-insoluble LR^a^ [%]	char [%]	total Yield [%]	alcohols [mg]	esters [mg]	aldehydes [mg]
1	blank	6.9	48.4		33.6	89	138.2	26.8	30.6
2	MgAlOx	2.5	50.0	20.7	2	75	679.9	194.2	169.3
3	CuO	12.7	53.5	8.0	1.3	76	254.5	61.2	29.2
4	Mn_2_O_3_	3.9	68.1	6.2	38	116	608.5	71.0	9.6
5	Cu_20_MgAlO_*x*_	14.8	91.7	18.4	0	125	3129	1615	290
6	Cu_20_Mn_2_MgAlO_*x*_	24.1	110.4	16.9	0	151	2516	1310	340
7	Cu_20_Mn_7_MgAlO_*x*_	28.1	87.0	17.0	0	132	2450	929	230
8	Cu_20_Mn_17_MgAlO_*x*_	33.9	85.5	16.3	0	136	2375	1458	210
9	Cu_8_Mn_17_MgAlO_*x*_	21.5	84.4	9.6	0	116	743	448	180
10	Mn_17_MgAlO_*x*_	8.7	59.4	7.4	0	75	285	185	190
11	5 wt % Pd/C	10.6	60.5	b	b	82	439	8.6	50
12	5 wt % Pt/C	11.4	67.2	b	b	88	332	70.2	66

a Lignin residue.

b For Pt/C and Pd/C, a distinction between THF-insoluble LR and Char was not included due to the carbon support interfering with numbers. Numbers are included in the total yield

**Figure 5:**
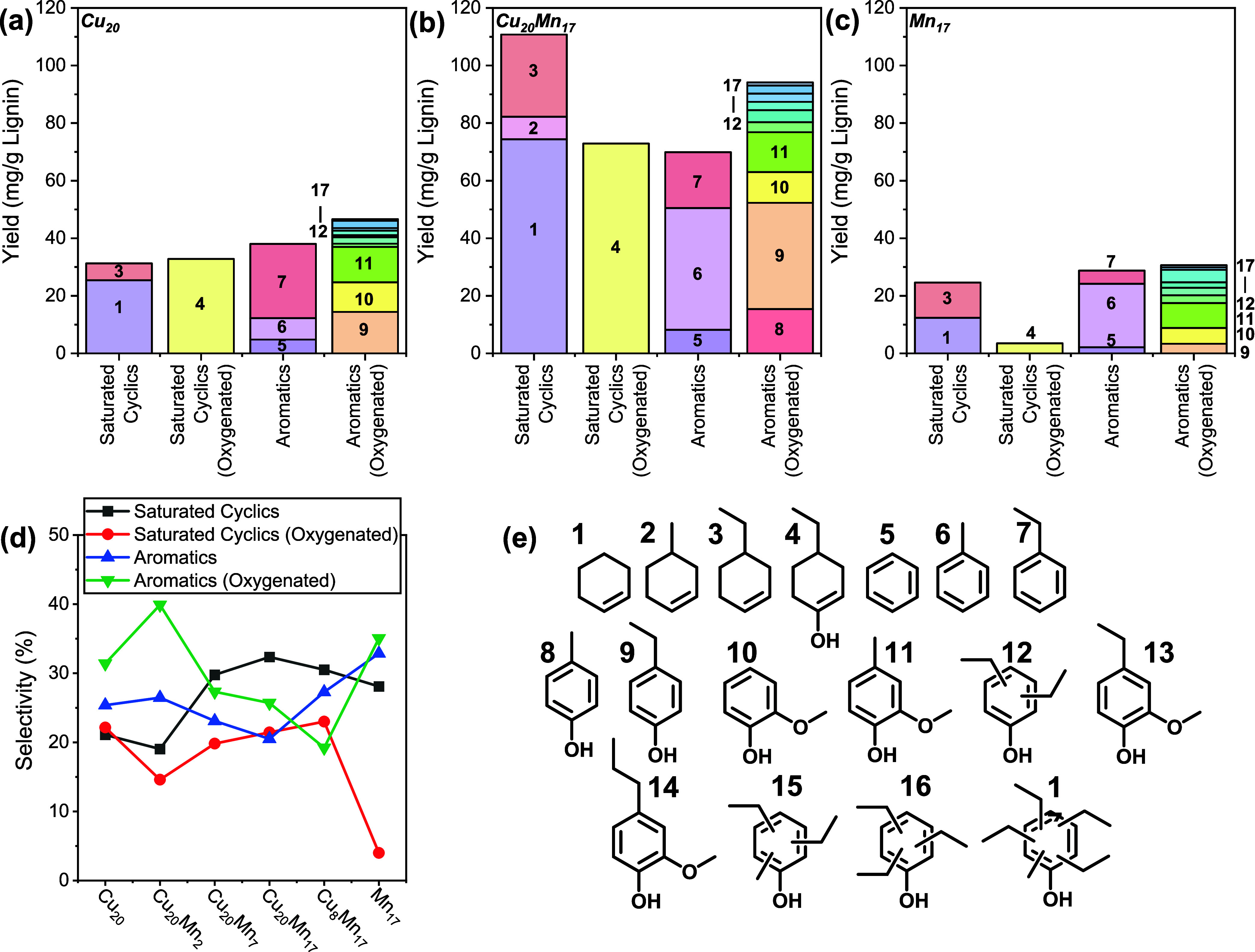
Monomer yield for selected CuMn-containing catalysts (a–c), the selectivity of catalysts (d), and the structure of the observed monomers (e) (1 g P1000, 0.5 g catalyst, 40 mL ethanol, 340 °C, 10 bar N_2_, 4 h).

Figure 5 shows the monomer yield and product distribution for a selection of catalysts, while Figure 5e shows the structure of observed monomers. The Cu_20_PMO and Mn_17_PMO catalysts are benchmarks against the optimum Cu_20_Mn_17_PMO catalyst in terms of monomer yield. For Cu_20_PMO, a wide range of monomers was observed, such as aromatics, cyclohexenes, and oxygenated aromatics. Oxygenated phenolics and guaiacol-type aromatics (**8**–**17**) were the dominant reaction products. Most of the monomers were alkylated by ethanol solvent or alcohol products derived thereof. The yield of all monomer products was higher when Cu_20_Mn_17_PMO was used as the catalyst instead of Cu_20_PMO. The largest yield increase was observed for saturated monomers (i.e., **1, 3, 4**). The product distribution obtained with Mn_17_PMO was similar to that obtained with Cu_20_PMO, although the yield of saturated oxygenated monomers **(4)** was much lower. This can be reasonably attributed to the absence of Cu, which provides a strong hydrogenation functionality. Figure 5d compares the product distributions for the different catalysts as a function of the Mn loading. Increasing the Mn loading increases the selectivity of (partially) saturated monomers at the expense of oxygenated aromatics. The best-performing Cu_20_Mn_17_PMO catalyst exhibits the highest selectivity of saturated monomers, next to the highest overall yield and degree of lignin solvolysis. The increased activity and selectivity toward saturated monomers is attributed to the oxophilic MnO species in the CuMnPMO. This aids in cleavage of lignin oligomers and hydrogenation reactions by Cu through increased activation of C=O bonds. The selectivity of aromatics and oxygenated saturated monomers for the optimum Cu_20_Mn_17_PMO sample was similar to that of the Cu_20_PMO benchmark.

**Figure 6:**
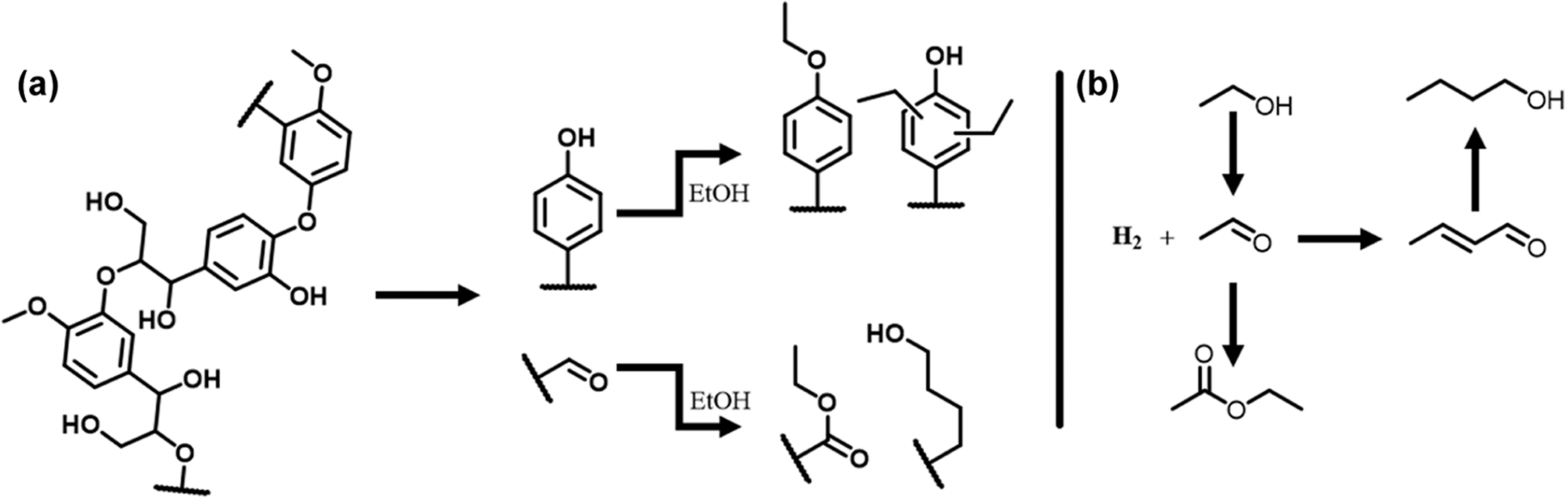
Simplified description of stabilization pathways occurring during catalytic solvolysis (a), and solvent conversion through the Guerbet reaction (b).

As earlier demonstrated by Huang et al.,^9^ reactive lignin fragments can be stabilized by capping with ethanol, as illustrated in Figure 6a. Huang et al. showed that basic Mg sites play an important in the capping of aliphatic/phenolic OH groups and aldehydes through Guerbet-type reactions. Figure 6b shows the mechanism of the Guerbet reaction.^9,28^ Increased activity in the Guerbet reaction aids in scavenging reactive formaldehyde species, hindering the repolymerization of lignin oligomers.^10^ Further, the esterification of reactive alcohol and aldehyde groups through Guerbet-type reactions also actively hinders repolymerization. Additionally, the Lewis acidic Al sites with ethanol are involved in capping phenolic OH groups and the alkylation of the aromatic ring. Limiting recondensation reactions through these side reactions is critical to the effective solvolysis of lignin. A drawback of the Guerbet activity of these catalysts is the conversion of the ethanol solvent to higher alcohols.^28^Table 3 also presents the yield of Guerbet products obtained from the ethanol solvent. The main reaction products are butanol and hexanol, while esters were formed through reactions of ethanol and butanol and hexanol through an aldehyde intermediate, resulting in ethyl hexanoate and ethyl butanoate, respectively. Acetaldehyde is the main aldehyde product observed, which can be formed through dehydrogenation on metallic Cu or hydrogen transfer to lignin or its reaction products. The Cu_20_PMO catalyst produced the largest amount of Guerbet products. The increasing amounts of Mn led to a decreasing yield of Guerbet products. Among the Cu-containing samples, Cu_20_Mn_7_PMO led to the smallest amount of Guerbet products. As expected, the Mn_17_PMO sample without Cu formed the least Guerbet products. Thus, an additional benefit of the addition of Mn is that it leads to a lower solvent conversion to less valuable side-products by decreasing the Mg content of the catalyst. Besides monomer yield, limiting solvent consumption is an additional benefit of doping Mn. Solvent consumption can be considered an important performance parameter.^29^ Furthermore, Mn was not found to affect the stabilization of reactive lignin intermediates by Cu.

Table 4 shows the average number molecular weight (*M*_n_) and average molecular weight (*M*_w_) calculated from the size exclusion chromatography traces, which are shown in Figure S3. SEC separates the compounds in the mixtures based on molecular weight, with calibration provided by a universal (polystyrene) standard. The P1000 starting lignin had an *M*_w_ of ∼3000 g/mol. The *M*_w_ of the lignin oils processed by the Cu-containing catalysts ranged from 1400 to 1700 g/mol, representing a substantial reduction in the molecular weight. The lowest molecular weight was achieved with the Cu_20_Mn_2_ catalysts. An increase of the Mn content led to a slight increase of *M*_w_, which we relate to the increased degree of alkylation. With a lower Cu content, Cu_8_Mn_17_ is less effective in depolymerization with a *M*_w_ of 1932 g/mol. The Mn_17_PMO catalyst without Cu had a slightly lower *M*_w_ of 1617 g/mol, although it must be noted that solubilization of the starting lignin was incomplete, which biases the *M*_w_ measurement. Thus, it can be said that Cu contributes significantly to the depolymerization reactions. Mn does not limit the alkylation ability of Cu and increases the rate of alkylation with increasing Mn weight loading, which leads to a higher *M*_w_.

**Table 4:** Average Number-Weight/Molecular Weight and the Polydispersity Index (PDI) Calculated from Size Exclusion Chromatography

	*M*_n_ (g/mol)	*M*_w_(g/mol)	PDI
P1000	1746	2987	1.65
Cu_20_	843	1663	1.97
Cu_20_Mn_2_	749	1415	1.88
Cu_20_Mn_7_	774	1617	2.00
Cu_20_Mn_17_	872	1701	1.95
Cu_8_Mn_17_	906	1932	2.13
Mn_17_	859	1617	1.88

**Figure 7:**
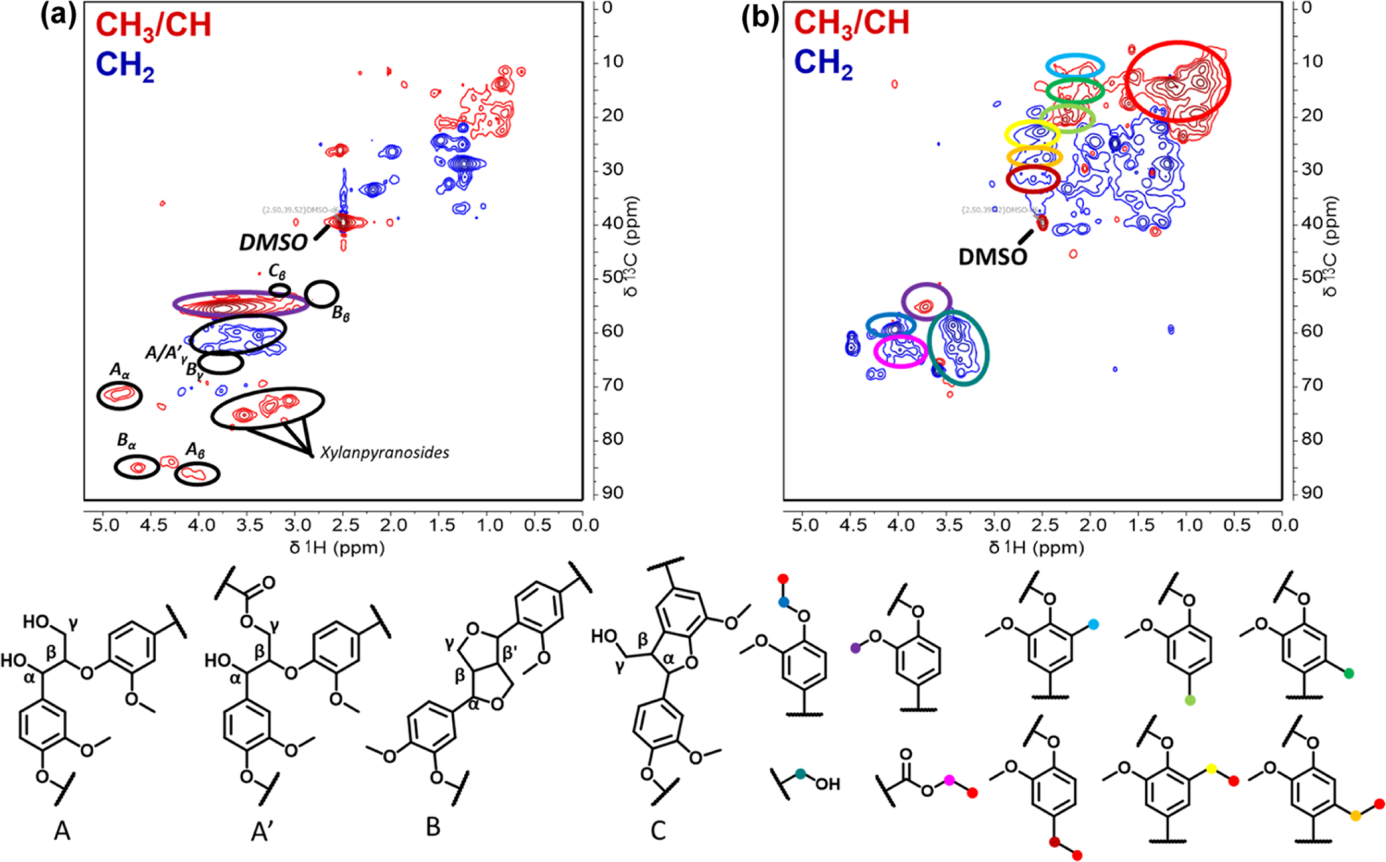
^1^H–^13^C gradient-edited HSQC of P1000 (a), and Lignin bio-oil obtained from Cu_20_Mn_17_PMO (b).

Figure 7a shows the structural characterization of P1000 lignin by ^1^H–^13^C HSQC NMR, highlighting the presence of characteristic lignin linkages below the image, such as β–β, β–O–4, and β–5. Features assignable to xylan pyranosides point to the presence of carbohydrates in the lignin. Figure S4 shows the aromatic region of the carbon-13 nuclear magnetic resonance (^13^C NMR) dimension of the HSQC NMR spectra, allowing for the semiquantitative determination of the ratio between syringyl (S), guaiacyl (G), and *p*-hydroxyphenyl (H) units. The ratio is determined based on the area of the individual H/G/S areas against the sum of all aromatic groups.^30^ The S/G/H ratio of P1000 lignin was thus determined to be 40:48:12. Visible for the starting lignin also the typical lignin linkers were observed and are displayed below Figure 7a, such as β–O–4 (A, A′), β–β (B), and β–5 (C).

Figure 7b shows the NMR spectra of the bio-oil obtained by solvolysis with the best-performing Cu_20_Mn_17_ catalysts, and some assignments are color-coded below the figure. NMR spectra for the bio-oils obtained with Cu_20_ and Mn_17_ are given in Figure S5. From these data, we obtained S/G/H ratios of 2:6:92 for Cu_20_PMO and 0:23:97 for Cu_20_Mn_17_PMO. These changes correspond to strong demethoxylation of the reaction products in the bio-oil, which is also supported by the decrease in the signal for methoxy groups (δC 55 ppm, δH 3.75 ppm). These lignin linkages were cleaved postsolvolysis for all tested catalysts. Instead, in this region, new peaks were assigned to ethoxy groups, ethyl esters, and primary alcohols. Notably, peaks commonly assigned to aldehydes were not observed. This confirms that capping occurred, involving ethanol and reactive oxygen species. Additionally, in the region between δC(10–40 ppm) and δH(0.5–3 ppm), methyl- and ethyl-alkyl units were found. The ethyl-alkyl units are derived from ethanol, while the methyl units are formed through in situ methanol formation via demethoxylation reactions. This further confirms the alkylation of lignin oligomers, besides the higher alkylated monomers observed through GC-MS analysis. The NMR of the bio-oil shows the stabilization of reactive oxygen intermediates by the CuMnMgAlO_*x*_ system for the effective depolymerization of technical lignin.

**Figure 8:**
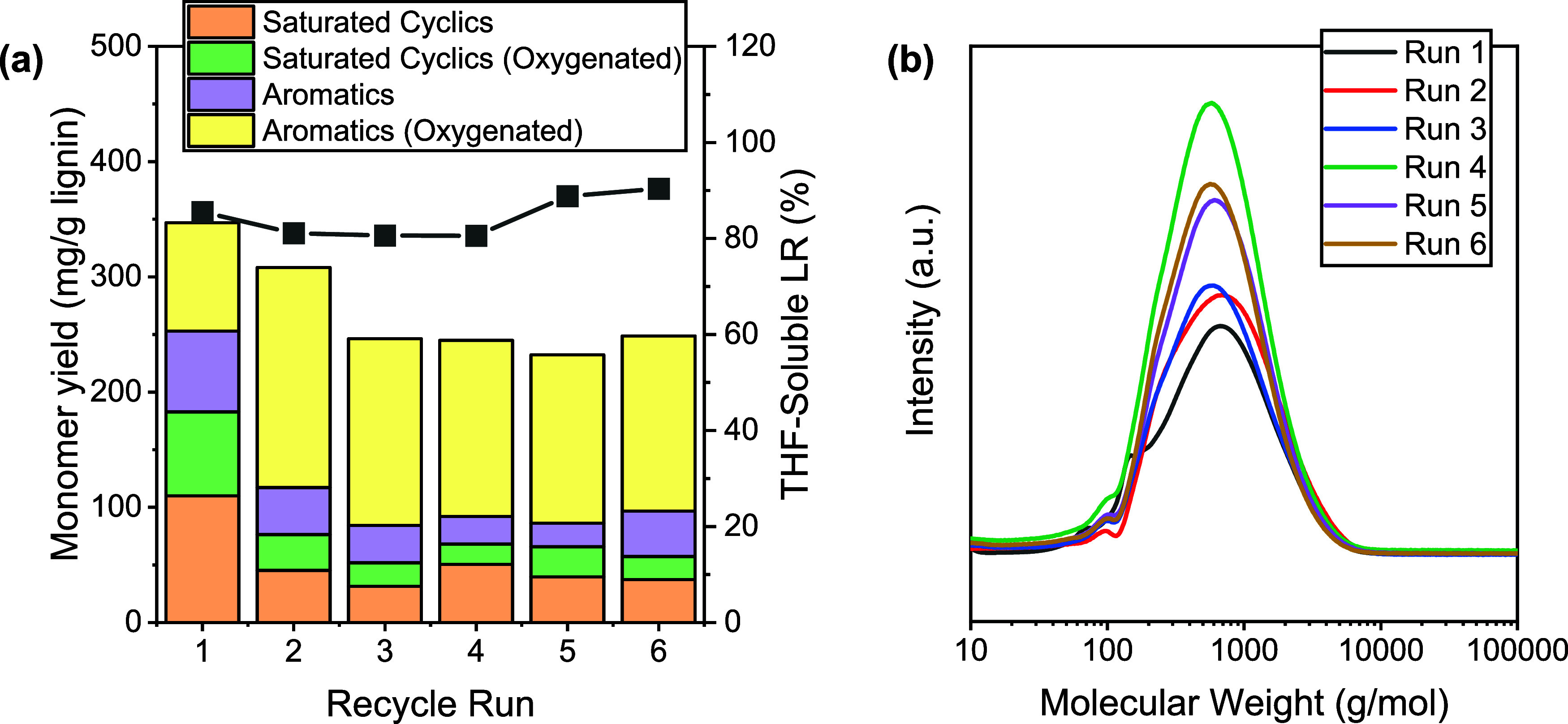
Recycle test of Cu_20_Mn_17_MgAlOx (a) and size exclusion chromatograms of the bio-oils obtained after each recycle run (b) (1 g P1000 lignin, 0.5 g catalyst, 40 mL ethanol, 340 °C, 10 bar N_2_, 4 h).

Several recycle tests were performed using the best-performing catalyst, Cu_20_Mn_17_MgAlO_*x*_, with P1000 in supercritical ethanol. Figure 8a shows the monomer yield for the six recycle tests. The catalyst was recovered after each reaction cycle and washed with THF before reusing. In the fresh state, the catalysts gave a monomer yield of 32.5%. Catalyst recycling led to a slight decline in monomer yield after two cycles. After three recycle runs, the monomer yield was approximately 25%. This decline is most likely due to the sintering of Cu at the reaction temperature of 340 °C. Although no char formation was observed on the catalyst, “THF-insoluble” lignin fractions can accumulate on the catalyst surface. This accumulation could contribute to blocking the active site, decreasing the catalytic activity. After three recycle runs, the monomer yield stabilized at around 25%. Figure 8b shows the SEC traces for each recycle run. The molecular weight of the bio-oil obtained after each reaction cycle was determined via SEC analysis. The calculated molecular weight determined from the SEC chromatograms indicated an average of ∼1800 ± 261 g/mol. This shows that while the monomer extraction efficiency decreased slightly with repeated catalyst recycling, the depolymerization activity remained consistently high over 6 runs.

## Conclusions

CuMgAlOx porous mixed oxides were doped with first-row transition metals by coprecipitation and calcination of precursor LDHs. Among the calcined CuPMO samples, the Mn-doped sample stood out regarding monomer and bio-oil yield obtained by catalytic solvolysis of P1000 alkaline lignin. At higher Mn content, a Cu–Mn spinel oxide was obtained, and the close proximity of Cu and Mn was confirmed by XRD and XPS analysis. Under reducing conditions, the predominantly Cu^2+^ and Mn^3+^ species were reduced to respectively Cu^0^ and Mn^2+^ in the active catalyst. The synergy between Cu^0^ and MnO likely underlies the enhanced efficiency in lignin solvolysis compared to unpromoted CuPMO. For all Mn-promoted CuPMO samples, complete solubilization of the lignin was achieved. An optimal yield of monomers was found in a catalyst containing a Cu/Mn ratio of unity. This catalyst could achieve a 2-fold increase in monomer yield from P1000 lignin compared to CuPMO. Increasing Mn content reduced solvent consumption, improving the overall atom efficiency of the process. With increasing Mn content, the selectivity shifted toward more saturated products, highlighting the enhanced hydrogenation activity of the mixed CuMn catalyst. Recycling the Cu_20_Mn_17_MgAlOx catalyst in supercritical ethanol resulted in a slight decline in monomer yield from 32.5% to approximately 25% after six runs while maintaining a high depolymerization activity.

## Supporting Information Available

additional Mg 1s/Al 2s XPS spectra; size exclusion chromatography traces of lignin bio-oil; HSQC NMR of lignin bio-oil; MS data of N_2_O chemisorption; and literature overview of catalytic solvolysis of technical lignin (PDF)
