# Supplementary material for: Mixed Cu–Mn Oxide Catalysts for Solvolysis of Technical Lignin
Source: ACS Sustain Chem Eng. 2025 Feb 18;13(8):3269–79. doi: 10.1021/acssuschemeng.4c09666 (PMC11881519; doi:10.1021/acssuschemeng.4c09666)
Supplement: Supplementary file 1 — sc4c09666_si_001.pdf [file sc4c09666_si_001.pdf]

Supporting Information

to

Mixed Cu-Mn oxide catalysts for solvolysis of technical lignin

Davey F. de Waard, Panos D. Kouris, Michael D. Boot, Emiel J.M. Hensen*

Laboratory of Inorganic Materials & Catalysis, Department of Chemical Engineering and Chemistry, P.O. Box 513, 5600 MB Eindhoven, Eindhoven University of Technology, The Netherlands

Corresponding authors:

Emiel J.M. Hensen

E-mail: e.j.m.hensen@tue.nl

Number of pages: 6

Number of Figures: 6

Number of Tables: 1

Contents

Figure S1: XPS spectra of selected CuMn PMOs: Mg 1s and Al 2s spectra.

Figure S2: XPS spectra of the first-row transition metal-doped PMOs: Mg 1s and Al 2s spectra.

Figure S3: Molecular weight distributions determined by size exclusion chromatography of product oils obtained by catalytic solvolysis using different catalysts.

Figure S4: Aromatics region of the ^{1}H-^{13}C HSQC NMR spectra of the bio-oil after solvolysis.

Figure S5: ^{1}H-^{13}C HSQC NMR spectra of the aliphatic region of the bio-oil after solvolysis.

Figure S6: MS data for N_2O chemisorption experiments.

Table S1: Catalytic solvolysis for technical lignin from literature focusing on temperature, atmosphere, batch time, and the monomer yield.

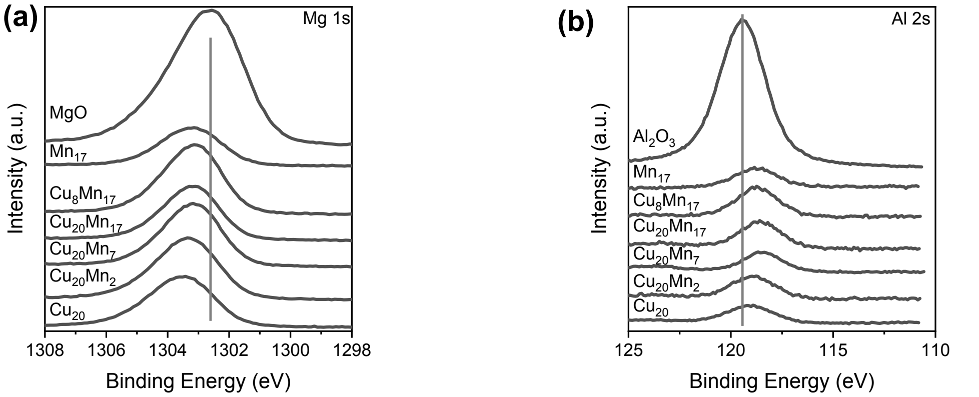

Figure S1: XPS spectra of selected PMOs: Mg 1s (a) and Al 2s (b) spectra.

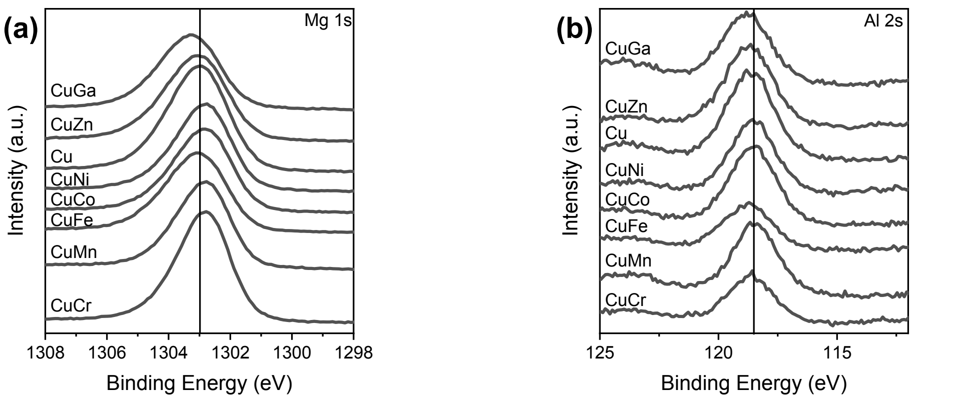

Figure S2: XPS spectra of the first-row transition metal-doped PMOs: Mg 1s (a) and Al 2s (b) spectra.

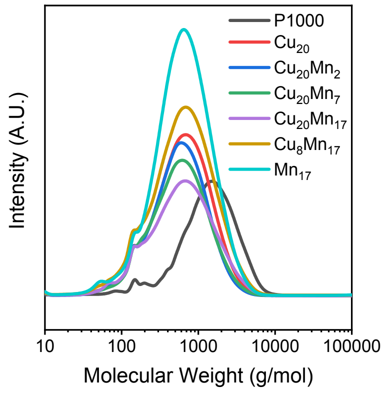

Figure S3: Molecular weight distributions determined by size exclusion chromatography of product oils obtained by catalytic solvolysis using different catalysts (1 g P1000, 0.5 g catalyst, 40 mL ethanol, 340°C, 10 bar N_2, 4 h).

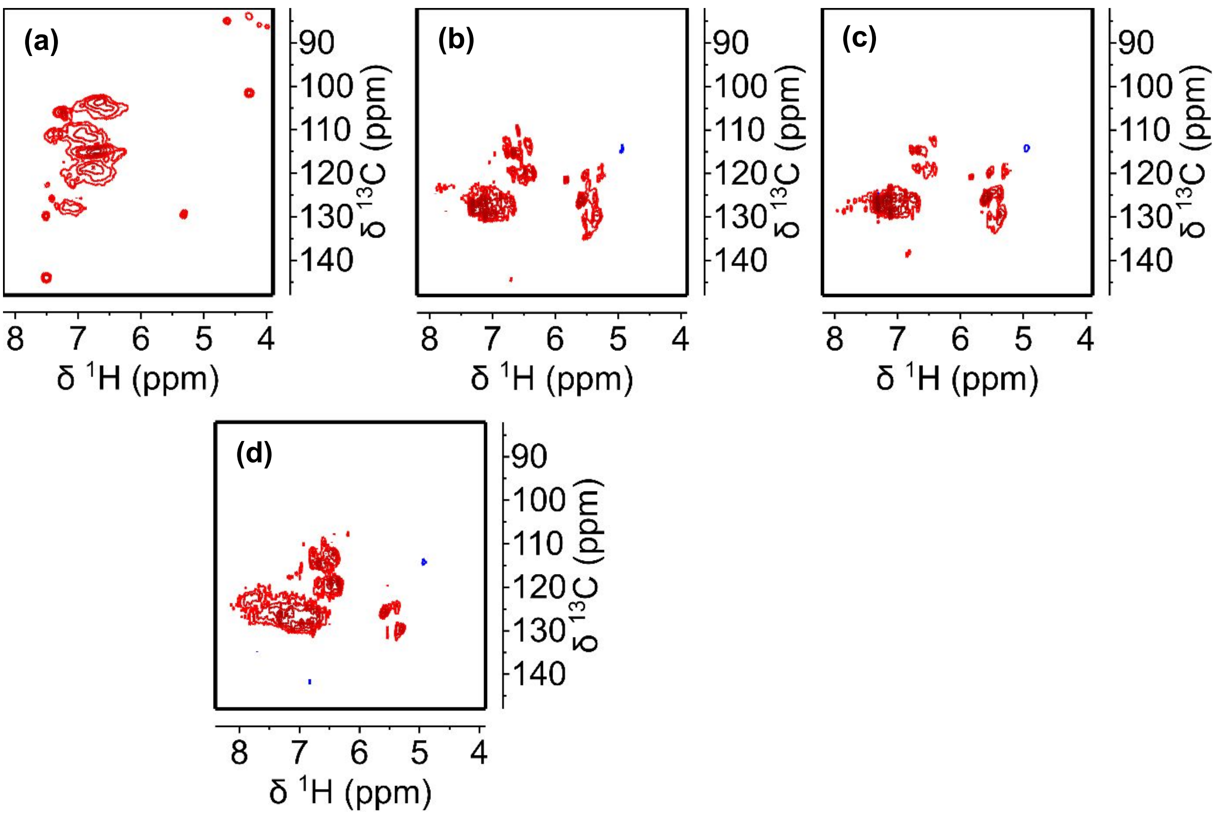

Figure S4: Aromatics region of the ^1H-^{13}C HSQC NMR spectra of the bio-oil after solvolysis of P1000 lignin (a), $Cu_{20}MgAlO_x$ (b), $Cu_{20}Mn_{17}MgAlO_x$ (c), and $Mn_{17}MgAlO_x$ (d). (1 g P1000, 0.5 g catalyst, 40 mL ethanol, 340°C, 10 bar N_2, 4 h).

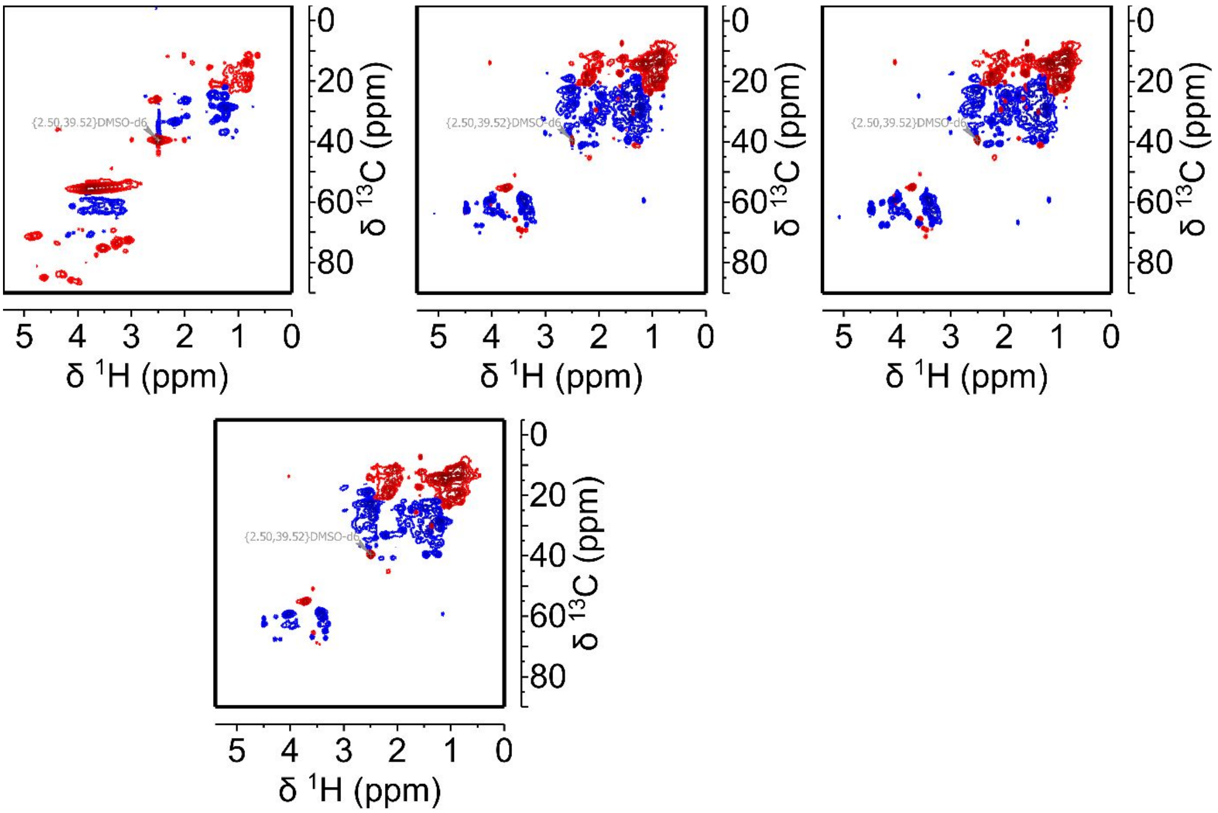

Figure S5: ^{1}H-^{13}C HSQC NMR spectra of the aliphatic region of the bio-oil after solvolysis of P1000 lignin (a), $Cu_{20}MgAlO_x$ (b), $Cu_{20}Mn_{17}MgAlO_x$ (c), and $Mn_{17}MgAlO_x$ (d). (1 g P1000, 0.5 g catalyst, 40 mL ethanol, 340°C, 10 bar N_2, 4 h).

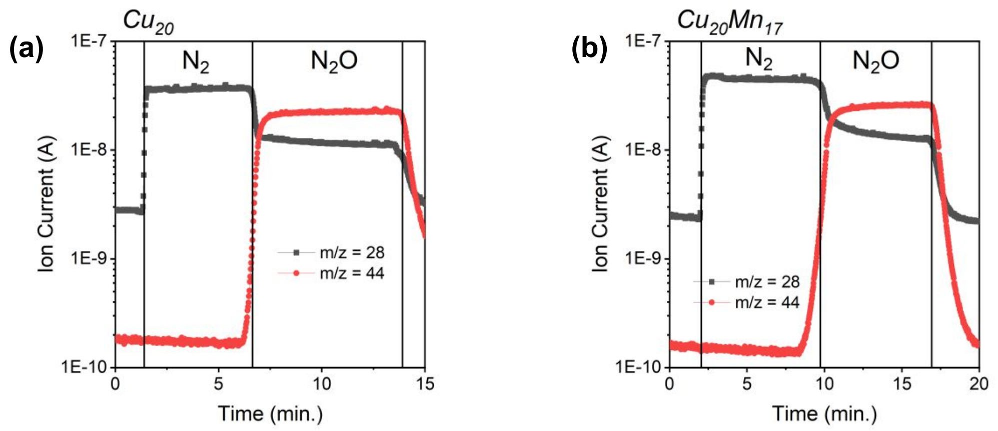

Figure S6: N_2O chemisorption experiments for $Cu_{20}MgAlOx$ (a), and $Cu_{20}Mn_{17}MgAlOx$ (b).

Table S1: Catalytic solvolysis for technical lignin from literature focusing on temperature, atmosphere, batch time, and the monomer yield.

Entry	Catalyst	Lignin	Solvent	Temperature [°C]	Initial Atmosphere	Time [hours]	Monomer Yield [wt%]	Ref.
1	Ru/C	Kraft	Ethanol	250	10 bar H_2	4	4.7	[1]
	Pt/C	Kraft	Ethanol	250	10 bar H_2	4	5.1	[1]
	Ni/C	Kraft	Ethanol	250	10 bar H_2	4	4.1	[1]
2	Pt/Al_2O_3	Kraft	No solvent	450	100 bar H_2	4	28	[2]
	Rh/Al_2O_3	Kraft	No solvent	450	100 bar H_2	4	30	[2]
3	$MgNiO_x/TiO_2$	Kraft	Dioxane/Methanol	320	20 bar H_2	24	22	[3]
4	PdCu/y-Al_2O_3	Soda	Ethanol/Water	200	10 bar H_2	6	13	[4]
	Pd/SiO2	Soda	Ethanol/Water	200	10 bar H_2	6	14	[4]
5	5Ni/HZSM-5	Organosolv	n-Butanol	300	20 bar H_2	4	19.5	[5]
6	3Pd/C	Organosolv	Ethanol	300	50 bar H_2	1	12	[6]
7	Ni + NaOH	Enzymatic Hydrolysis Lignin	Ethylene glycol	200	30 bar H_2	6	18.8	[7]
8	Ni/Al-SBA-15	Hydrolysis lignin	Ethanol	280	10 bar H_2	4	17.8	[8]
9	CuMnMgAlOx	Soda	Ethanol	340	10 bar N_2	4	34	This work

References

(1) Bartolomei, E.; Le Brech, Y.; Gadiou, R.; Bertaud, F.; Leclerc, S.; Vidal, L.; Le Meins, J. M.; Dufour, A. Depolymerization of Technical Lignins in Supercritical Ethanol: Effects of Lignin Structure and Catalyst. *Energy and Fuels* **2021**, *35* (21), 17769–17783. https://doi.org/10.1021/acs.energyfuels.1c02704.

(2) Hita, I.; Deuss, P. J.; Bonura, G.; Frusteri, F.; Heeres, H. J. Biobased Chemicals from the Catalytic Depolymerization of Kraft Lignin Using Supported Noble Metal-Based Catalysts. *Fuel Processing Technology* **2018**, *179*, 143–153. https://doi.org/10.1016/J.FUPROC.2018.06.018.

(3) Zhang, X.; Li, W.; Wang, J.; Zhang, B.; Guo, G.; Shen, C.; Jiang, Y. Depolymerization of Kraft Lignin into Liquid Fuels over a WO3 Modified Acid-Base Coupled Hydrogenation Catalyst. *Fuel* **2022**, *323*, 124428. https://doi.org/10.1016/J.FUEL.2022.124428.

(4) Atanasova, B.; De Saegher, T.; Poelman, H.; de Reviere, A.; Vercammen, J.; Verberckmoes, A.; De Clercq, J.; Lauwaert, J. Pd Catalysts in the Mild Reductive Depolymerization of Soda Lignin: Support and Cu Addition Effects. *Chemical Engineering Journal* **2024**, *498*, 155866. https://doi.org/10.1016/J.CEJ.2024.155866.

(5) Liu, X.; Jiang, Z.; Feng, S.; Zhang, H.; Li, J.; Hu, C. Catalytic Depolymerization of Organosolv Lignin to Phenolic Monomers and Low Molecular Weight Oligomers. *Fuel* **2019**, *244*, 247–257. https://doi.org/10.1016/J.FUEL.2019.01.117.

(6) Hakonen, K. J.; González Escobedo, J. L.; Meriö-Talvio, H.; Hashmi, S. F.; Karinen, R. S.; Lehtonen, J. Ethanol Organosolv Lignin Depolymerization with Hydrogen over a Pd/C Catalyst. *ChemistrySelect* **2018**, *3* (6), 1761–1771. https://doi.org/10.1002/SLCT.201702701.

(7) Sang, Y.; Ma, Y.; Li, G.; Cui, K.; Yang, M.; Chen, H.; Li, Y. Enzymatic Hydrolysis Lignin Dissolution and Low-Temperature Solvolysis in Ethylene Glycol. *Chemical Engineering Journal* **2023**, *463*, 142256. https://doi.org/10.1016/J.CEJ.2023.142256.

(8) Chen, P.; Zhang, Q.; Shu, R.; Xu, Y.; Ma, L.; Wang, T. Catalytic Depolymerization of the Hydrolyzed Lignin over Mesoporous Catalysts. *Bioresour Technol* **2017**, *226*, 125–131. https://doi.org/10.1016/J.BIORTECH.2016.12.030.
